# Trajectories of mindfulness, flow experience, and stress during an online-based MBSR program: the moderating role of emotional exhaustion

**DOI:** 10.3389/fpsyg.2024.1385372

**Published:** 2024-07-15

**Authors:** Charlotte Hohnemann, Florian Engel, Corinna Peifer, Stefan Diestel

**Affiliations:** ^1^Work, Organizational, and Business Psychology, Bergische Universität Wuppertal, Wuppertal, Germany; ^2^Work and Organizational Psychology, Ruhr-Universität Bochum, Bochum, Germany; ^3^Work and Organizational Psychology, Universität zu Lübeck, Lübeck, Germany

**Keywords:** mindfulness-based stress reduction, flow experience, perceived stress, change trajectories, emotional exhaustion

## Abstract

**Introduction:**

Despite numerous papers focusing on mindfulness at work, our knowledge about how flow experience and stress as indicators of optimal functioning and wellbeing at work evolve over time during the common mindfulness-based stress reduction (MBSR) program remains limited. Drawing from the transactional model of flow and stress, we argue that a build-up of mindfulness over the training duration not only leads to a decrease in stress but also an increase in flow experience. Thereby, we examine the moderating role of emotional exhaustion amplifying the beneficial effects of mindfulness.

**Methods:**

In a quasi-experimental study, 91 participants completed weekly questionnaires over the course of 8 weeks. Forty six participants in the experimental group took part in the MBSR program, while 45 participants were part of an inactive control group.

**Results:**

Mindfulness and flow showed a significant linear increase over time, whereas stress exhibited a linear decrease. Those who participated in the MBSR training reported an increase in mindfulness that positively and negatively predicted the trajectories of flow and stress, respectively. Emotional exhaustion amplified the effects of the trajectory of mindfulness on the trajectories of flow and stress.

**Discussion:**

These findings suggest that mindfulness can not only reduce stress but can also foster the autotelic experience of flow, especially for chronically depleted individuals. However, more research is necessary to replicate these results and address the limitations of the current study, including the quasi-experimental design, the use of self-report measures, as well as the dropout during the study period.

## 1 Introduction

In the literature on stress and wellbeing, the concept of mindfulness has become a rising and staying star. Core topics in this research tradition have centered around the question of how mindfulness can be systematically fostered by specific training and interventions to enhance mental wellbeing (e.g., Jamieson and Tuckey, 2017; Bartlett et al., 2019). In particular, the mindfulness-based stress reduction program (MBSR) (Kabat-Zinn, 2003) has been successfully applied to reduce stress in the workplace (Khoury et al., 2015; Vibe et al., 2017). However, to craft a more enriching work experience that concurrently enhances performance and wellbeing, it is essential to shift the focus from stress reduction alone to the promotion of optimal functioning. One of the most important indicators of optimal psychological functioning at work is flow experience, which is characterized by an engrossing experience during intense concentration on the current task and therefore fosters wellbeing and performance simultaneously (Csikszentmihalyi, 1975). Previous research about the positive effects of mindfulness on wellbeing and motivation suggests that MBSR training could be utilized beyond a reduction of stress and foster optimal psychological functioning in the workplace (Khoury et al., 2015; Vibe et al., 2017). However, prior studies assessing the effects of mindfulness on flow experience have created mixed results (Sheldon et al., 2015). Hence, despite these potential benefits of mindfulness at work to foster flow experience and the widespread application of the MBSR training off-the-job and within companies (Grossman et al., 2004; Bartlett et al., 2019), our knowledge of whether the MBSR program can successfully enhance optimal experience in the work context, as indicated by flow experience, remains limited.

Even though scholars provided initial evidence that mindful individuals also tend to experience more flow (Kee and Wang, 2008; Moore, 2013), research also showed that mindful perception of the situation and the absorption during flow cannot be experienced simultaneously (Sheldon et al., 2015). Addressing this paradox, we focus on how a build-up in mindfulness over the training duration of several weeks can influence flow and stress. In doing so, we delineate our research model based on the transactional model of stress and flow (TSF) (Peifer and Tan, 2021), which extends the traditional transactional model of stress and coping (Lazarus and Folkman, 1984). In particular, we argue that a build-up of mindfulness over the training duration of the MBSR training enables an increase in flow experience along with a decrease in stress. During the training, participants develop a clearer and more accepting perception of demanding situations as well as more efficient resource allocation (Glomb et al., 2011; Good et al., 2016), which facilitates flow and reduces stress as work situations are perceived as positive challenges rather than stressors (Lazarus and Folkman, 1984; Peifer and Wolters, 2021). Accordingly, with a weekly assessment of mindfulness, flow experience, and stress during the MBSR training and an inactive control group, we examine the change trajectories in these variables as well as their relations. In doing so, we extend previous research about the effects of the MBSR training that has mainly focused on the comparison between pre- and post-measurement (e.g., Bartlett et al., 2019; Dust et al., 2021; Lyddy et al., 2021).

**Figure 1:**
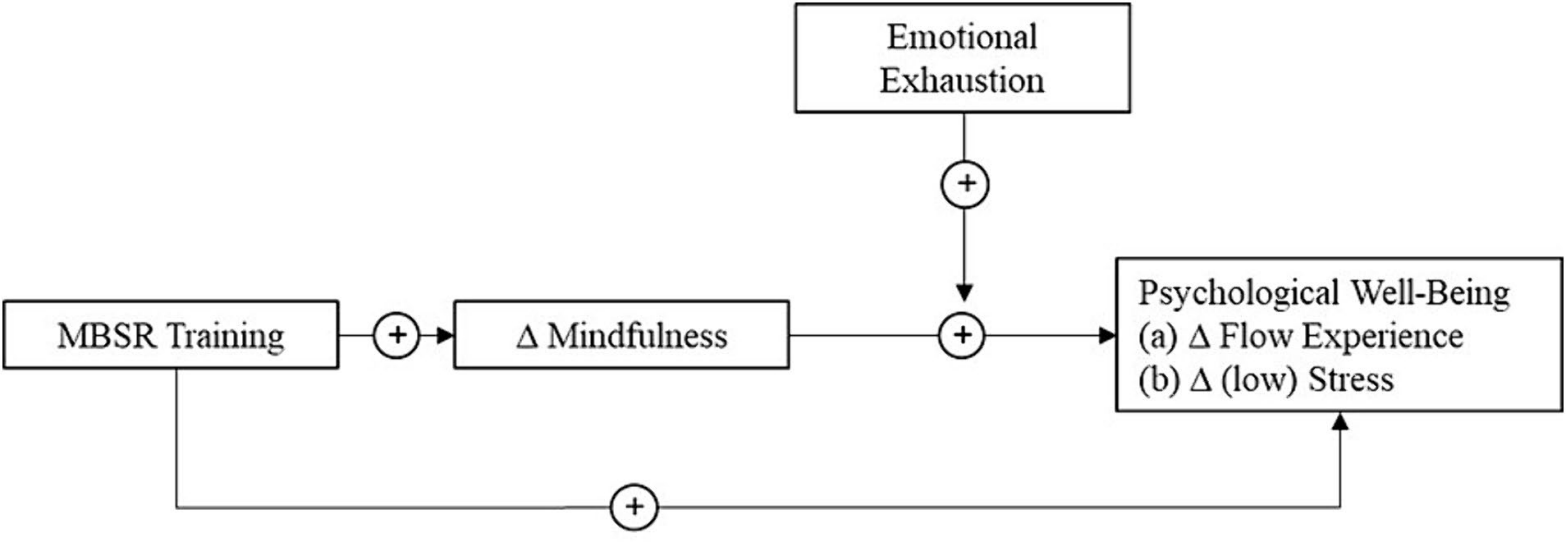
Theoretical model. The symbol ∆ indicates temporal changes (i.e., trajectories) in the corresponding variables during the mindfulness intervention.

Furthermore, the relations between the change trajectory of mindfulness and the trajectories of stress and flow experience at work are likely contingent upon the individual’s level of resources, indicated by emotional exhaustion, which refers to a chronic state of depleted emotional and physiological resources (Cropanzano et al., 2003). If resources are depleted at work, for instance by job demands, and individuals show high emotional exhaustion, a more efficient allocation of remaining resources is especially important to prevent their interpretation as a stressor but positive challenge. Accordingly, we hypothesize emotional exhaustion to moderate (i.e., strengthen) the positive association between changes in mindfulness and changes in both stress and flow experiences. Figure 1 depicts our conceptual model.

In summary, our study extends the current state of research in at least three ways. Firstly, by focusing on a timeframe of several weeks, we provide new insights into whether mindfulness and related interventions can be utilized to promote flow experiences as critical indicators of optimal functioning at work. Despite certain dimensions of mindfulness and flow appearing incompatible (Sheldon et al., 2015), we contribute to the existing literature on workplace interventions by exploring the potential of the MBSR training to enhance flow experience, thereby opening up new opportunities for cultivating optimal functioning within the workplace. In doing so, our research not only enhances our understanding of the relations between mindfulness and flow but also holds promise for improving wellbeing and performance in work environments.

Second, while previous studies have primarily compared pre- and post-measurements of mindfulness interventions, our study takes a different approach by examining the shape of the trajectory of mindfulness, stress, and flow over time. By investigating the diverse change patterns, such as stable growth or quadratic trajectories, as well as their relationships, we offer new insights into the unfolding of the MBSR program and its effects on individuals in the workplace. This longitudinal perspective enables us to provide a deeper understanding that can inform the development and implementation of more effective mindfulness-based interventions.

Lastly, we highlight the role of interpersonal differences in emotional exhaustion when examining the benefits of mindfulness intervention on flow and stress. As recent evidence suggests that mindfulness-based interventions may not always facilitate psychological health (Farias et al., 2020; Britton et al., 2021), a thorough investigation of how chronically depleted resources interact with changes in mindfulness adds to our understanding of stress prevention and long-term wellbeing stabilization. In other words, we provide novel insights into the interplay of interpersonal boundary conditions and intra-personal changes in mindfulness in predicting employees’ wellbeing over time.

## 2 Effects of the MBSR training on flow and stress over time

Mindfulness is conceptualized as meta-cognitive awareness that involves an intentional observation of internal and external experiences at the present moment in an accepting and open manner (Good et al., 2016). By decoupling the self (e.g., self-concept, self-esteem, ego) from those experiences, individuals in a mindful state perceive their experiences neutrally (Glomb et al., 2011). The MBSR training is one of the most known mindfulness interventions that entails several mindfulness practices over the course of 8 weeks reducing stress, depression as well as anxiety and fostering (self-)compassion, empathy, and mental health (Eberth and Sedlmeier, 2012; Khoury et al., 2015; for further details about the training program see Santorelli et al., 2017). Considering mindfulness as an explaining mechanism, several reviews and meta-analyses comparing pre- and post-measurements support the claim that the MBSR training reduces stress (e.g., Khoury et al., 2015; Vibe et al., 2017; Bartlett et al., 2019). In contrast, previous studies yield mixed evidence for the effects of mindfulness on the engrossing experience of flow (Kee and Wang, 2008; Sheldon et al., 2015), while the effects of the MBSR training or similar mindfulness interventions have not been investigated yet. In particular, the distinct nature of complete absorption in flow contrasts with the open awareness encompassed in mindfulness (Sheldon et al., 2015). However, facets of mindfulness such as heightened attentional control and present-moment focus may facilitate the experience of flow (Kee and Wang, 2008; Moore, 2013). By resolving this discrepancy, we acknowledge that mindfulness and flow cannot be experienced simultaneously but anticipate that the development of mindfulness, including specific aspects like present-moment attention and sustained attention, throughout the multi-week MBSR training, will contribute to an increase in the frequency of flow experiences.

To further derive our predictions about how changes in mindfulness affect changes in stress and flow during the MBSR training, we draw on the TSF (Peifer and Tan, 2021), which integrates the transactional stress model (Lazarus and Folkman, 1984) and insights from the flow channel model (Csikszentmihalyi, 1975). These models highlight that a similar situation can be experienced as stressful or as positive challenge, depending on the individuals’ perception of demands and available resources. When the perceived demands exceed available resources, individuals may appraise the situation as a threat and experience stress or anxiety (Peifer and Tan, 2021). In contrast, individuals may view a situation as positive challenge and experience flow, when they perceive a high-level balance between those (Peifer and Tan, 2021). Integrating these notions from the TSF with previous research that has repeatedly shown that mindfulness can improve coping with stress and view demanding situations as positive challenges (Garland et al., 2017; Jamieson and Tuckey, 2017; Beer et al., 2020; Sun et al., 2020), we assume that the development of mindfulness during the MBSR training evokes a decrease in stress as well as an increase in flow. In particular, by gradually increasing attention to present-moment experiences and reducing the automatization of cognitive processes during the MBSR program, individuals can evaluate situations more composedly and be less influenced by emotions and automated thought patterns such as worrying. Hence, they can allocate cognitive resources more efficiently (Bishop et al., 2004; Glomb et al., 2011; Good et al., 2016). Further, with increasing mindfulness during the MBSR program, internal and external stimuli such as demands or emotions become more decoupled from initial judgments and observed in an accepting way (Glomb et al., 2011). Therefore, demanding aspects or situations are likely to be less threatening and intruding which reduces perceived stress over time (Beer et al., 2020). In contrast, individuals can concentrate all available resources on the task itself establishing a high-level balance between demands and skills and enabling more flow during the mindfulness training (Beer et al., 2020; Sun et al., 2020).

Despite the aim of the MBSR training to increase mindfulness and decrease stress over time, there is a scarcity of research investigating the specific patterns of change in these variables (Snippe et al., 2017; Andreotti et al., 2018). The MBSR training follows a structured program, which includes weekly training sessions with theoretical explanations and various mindfulness exercises (e.g., body scanning, and present-moment awareness; Santorelli et al., 2017). Participants develop and refine their mindfulness skills through these exercises, leading to a heightened state of mindfulness over time (Baer et al., 2012). Therefore, we expect a consistent and gradual increase in mindfulness, represented by a positive linear trajectory throughout the eight-week training duration. Supporting this prediction, Baer et al. (2012) found descriptive evidence for a linear increase in mindfulness over the training period. By integrating the anticipated linear changes in mindfulness with the established effects on stress and flow, we propose that increased mindfulness will lead to a similar linear increase in flow experience and a decrease in stress. Regarding stress, Snippe et al. (2017) supported a linear decrease over the entire training duration, whereas Baer et al. (2012) observed a (linear) change only after the third week. Positive indicators of optimal functioning, such as the autotelic experience of flow, have not been examined to date. Accordingly, based on notions of the TSF and supported by initial empirical evidence for linear change patterns (Baer et al., 2012; Snippe et al., 2017; Peifer and Tan, 2021), we put forward the following hypotheses:

*H*1a: Mindfulness exhibits a linear increase over the training duration.

*H*1b: Flow experience exhibits a linear increase over the training duration.

*H*1c: Stress exhibits a linear decrease over the training duration.

Linking our predictions on trajectories to the proposed effects of MBSR training and mindfulness on flow and stress, we derive the following hypotheses. Because we consider mindfulness as the core mechanism of the effects of the MBSR training (Pascoe et al., 2017; Vibe et al., 2017), we propose changes in mindfulness to mediate the effects of the MBSR training on changes in flow and stress.

*H*2: The linear increase in mindfulness positively predicts (a) the linear increase in flow and (b) the linear decrease in stress.

*H*3: The MBSR training positively predicts (a) the linear increase in flow and (b) the linear decrease in stress via the trajectory of mindfulness.

## 3 Moderating effects of emotional exhaustion

Moreover, the effects of changes in mindfulness on changes in flow and stress are likely dependent on the individual level of emotional exhaustion. Emotional exhaustion represents the core dimension of burnout and results from prolonged strain that reflects chronically depleted resources due to high work demands (Demerouti et al., 2001). Whereas emotional exhaustion represents a relatively persistent state that can last over longer time periods (Toppinen-Tanner et al., 2002), fatigue and stress vary largely within individuals and are strongly influenced by current demands (e.g., Baethge and Rigotti, 2013; Hülsheger, 2016). Individuals with high emotional exhaustion have a lower baseline level of available resources, making them more likely to perceive demanding work situations as stressors rather than positive challenges (Trougakos et al., 2015). Mindfulness can be especially helpful for individuals with low resource availability by promoting a clearer perception of the situation which is less influenced by negative experiences such as feeling overwhelmed (Good et al., 2016). Enhanced attentional control enables individuals to stay focused on the task at hand and overcome worries or negative emotions, which also facilitates a more efficient allocation of remaining resources (Glomb et al., 2011). Given the importance of overcoming negative states and effectively allocating limited resources for chronically depleted individuals, we expect the build-up of mindfulness over the training duration to exert stronger effects on changes in stress and flow experience for individuals with high emotional exhaustion compared to those with low emotional exhaustion. Accordingly, previous research has suggested that mindfulness is particularly beneficial for individuals with chronic feelings of depletion and recurring worries or negative moods (Baer, 2003; Creswell and Lindsay, 2014).

*H*4: Emotional exhaustion amplifies (a) the positive effects of the increase in mindfulness on the increase in flow and (b) the decrease in stress.

In conclusion, in the present study, we delineate and examine a moderated mediation model in which we predict that the MBSR program will lead to a linear increase in mindfulness which subsequently leads to a linear increase in flow experience as well as a linear decrease in stress. In addition, we propose that the positive effects of the increase in mindfulness on the increase in flow and the decrease in stress are amplified by individual levels of emotional exhaustion.

*H*5: Emotional exhaustion amplifies the indirect effects of the MBSR training on the increase in (a) flow and (b) the decrease in stress via the increase in mindfulness.

## 4 Materials and methods

### 4.1 Sample and procedure

We conducted a quasi-experimental study with an experimental group participating in online-based MBSR classes and an inactive control group. Ethical approval was obtained from the university’s ethical committee (masked for review). The experimental group consisted of participants who enrolled in certified German MBSR trainers’ online classes conforming to the standardized eight-week structure. We contacted all trainers who offered MBSR courses between April and June 2020 asking to forward the informational material to their participants. The inactive control group was recruited during the same time frame through social media and personal contacts. Inclusion criteria were age of at least 18 years and no regular engagement in mindfulness training. Participants in the control group received 15€ for completing the study.

**Figure 2:**
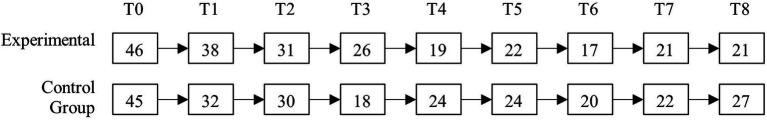
Sample sizes in the experimental and control group at each point of measurement. T0 indicates the pre-questionnaire. T1–T8 indicate weekly questionnaires over the training duration.

A total of 48 participants in the experimental group and 49 participants in the control group completed the initial questionnaire (T0). Two participants from the experimental group were excluded as their MBSR class was conducted in person, not online, for consistency among all groups. Four participants from the control group were excluded because they reported regular mindfulness practice.^1^ During the eight-week assessment period (T1–T8) following the pre-questionnaire, all participants received weekly questionnaires measuring mindfulness, flow experience, and stress. Figure 2 displays the final sample sizes for each questionnaire.

Participants included in the final analysis showed an average age of 39.98 years (SD = 14.77). Fifty nine participants were female (65%), whereas 32 participants were male (35%). Sixty eight percent of the participants were working, while 20% stated to be still in education (i.e., in school or university). The remaining participants took care of the household and family members (5%), were retired (5%), or were on job search (3%).

### 4.2 Measures

Mindfulness was measured retrospectively every week with the Cognitive and Affective Mindfulness Scale-Revised (CAMS-R) by Feldman et al. (2007). We translated the items from English into German based on the back-translation procedure (Brislin, 1970). The 12 items were rated on a scale ranging from 1 (almost never) to 4 (almost always) in relation to last week. An example item is “I could accept the thoughts and feelings I had.”

Flow experience was assessed retrospectively every week by 10 items of the flow frequency scale by Bartzik et al. (2021). The scale consists of three sub-facets absorption, perceived skill-demand-balance, and enjoyment. Items were rated on a scale from 1 (never) to 6 [(almost) always]. An example item is “In the last week, how often did you find yourself completely absorbed in an activity at work/study?”

Stress was measured retrospectively every week with the Irritation Scale by Mohr et al. (2005) which assesses emotional as well as cognitive strain in the work context. The concept of irritation captures a psychological stress reaction with a medium intensity which is less influenced by the fluctuations of work demands but has been shown to capture intraindividual fluctuations in strain (e.g., Dormann and Zapf, 2002; Baethge and Rigotti, 2013). The eight items were rated from 1 (I do not agree at all) to 7 (I completely agree) in relation to the last week. An example item is “I found it hard to detach myself after work.”

Emotional exhaustion was assessed during the pre-questionnaire with six items from the German version of the Maslach Burnout Inventory (MBI-D) (Maslach et al., 1986; Büssing and Perrar, 1992). Items were rated from 1 (this feeling/situation does not occur at all) to 6 (this feeling/situation occurs very often). An example item is “I feel burnt out by my work.”

### 4.3 Preliminary analysis

#### 4.3.1 Sample characteristics and response rate

The program R was used for all following analyses (Version 1.4) (R Core Team, 2019). We tested whether the experimental and control groups significantly differed in demographic characteristics, study variables during the pre-assessment, and their response rate. Independent t-tests revealed that the experimental group was older than the control group [*t*(88) = 3.50, *p* < 0.001; experimental group: M = 45.02, SD = 14.88; control group: M = 34.82, SD = 12.89] and experienced higher stress during the pre-assessment [*t*(87) = 2.33, *p* = 0.022; experimental group: M = 3.42, SD = 1.46; control group: M = 2.76, SD = 1.25]. All other variables (i.e., gender, emotional exhaustion, response rate, mindfulness, and flow experience during the pre-assessment) did not differ between both groups (all *p* > 0.06). In our analyses of the proposed between-person effects, we controlled for age, gender, as well as the baseline measure of mindfulness, flow experience, and stress to account for a possible influence on the examined trajectories.

#### 4.3.2 Construct validity

In order to ensure construct validity, second-order multilevel confirmatory factor analyses were conducted using Maximum Likelihood. The model with separate factors for the variables mindfulness, flow experience (consisting of its three sub-facets), stress (consisting of its two sub-facets) and emotional exhaustion provided the best fit [χ^2^(406) = 1597.19, *p* < 0.001; RMSEA = 0.078, 90% CI [0.074; 0.082]; SRMR (within) = 0.062, SRMR (between) = 0.025, CFI = 0.892]. Other models provided a worse fit [e.g., combining all sub-facets of flow and stress: χ^2^(408) = 1886.77, *p* < 0.001; RMSEA = 0.087, 90% CI [0.083; 0.091]; SRMR (within) = 0.114, SRMR (between) = 0.025, CFI = 0.866]. These results provide support that our measured variables represent distinct constructs.

#### 4.3.3 Intra class coefficients

Based on a Bayesian random-intercept model, we calculated Intra Class Coefficients (ICCs) for mindfulness (ICC = 0.29), flow (ICC = 0.23), and stress (ICC = 0.24) (Chen et al., 2011; Dust et al., 2021). These results support considerable intra-individual variation and subsequently our analysis of change trajectories over the measurement period.

### 4.4 Analytical strategy

When analyzing the individual change trajectories and their relation, we used Bayesian estimation because empirical Bayes estimates calculated for each participant are additionally weighted by overall sample information providing a more accurate representation than estimates based on separate regression models for each participant (Chen et al., 2011). Additionally, Bayesian estimation can successfully handle small sample sizes as well as non-normal and skewed posterior distributions, for instance in the case of indirect effects (Zyphur and Oswald, 2015). We relied on the Markov chain Monte Carlo method (MCMC) with non-informative priors because no studies have been conducted that can provide reliable baseline information on the relationships assessed in this study. In doing so, we allow the estimation of the posterior distribution of the parameters to be dominated by the collected data (Zyphur and Oswald, 2015). We followed suggestions by Depaoli and van de Schoot (2017) and assessed several indicators of Bayesian diagnostics to ensure the correct specification of our Bayesian models. Results support the correct convergence of our models, a limited influence of the non-informative prior, the credibility of posterior distributions, and acceptable autocorrelation. These results further suggest that the sample size was sufficient for our model estimation. For further information, please review the Supplementary material or contact the first author.

#### 4.4.1 Analyses of change trajectories (within-person level)

Following recommendations from previous studies (Chen et al., 2011; Dust et al., 2021), we calculated mixed models with a random intercept and fixed slope to examine the change trajectories of mindfulness, flow experience, and stress. Slopes were fixed among participants since we aimed to examine a general trajectory for the whole sample (Dust et al., 2021). We added time as a level 1 predictor, where the baseline measure equaled zero and week eight equaled eight. To rule out non-linearity, we also tested for quadratic and cubic slopes in all outcomes. Table 1 provides information about the parameter estimates.

**Table 1:** Means, standard deviation, correlations, and Cronbach’s alpha.

		*M*	*SD* (between)	*SD* (within)	1	2	3	4	5	6	7	8	9
**Within-variables**													
1.	Weekly mindfulness (T0–T8)	2.78	0.45	0.23	*0.80–0.88*	**0.62**	**−0.62**						
2.	Weekly flow experience (T0–T8)	3.83	1.09	0.52		*0.94–0.98*	**−0.42**						
3.	Weekly stress (T0–T8)	2.87	1.26	0.57			*0.84–0.92*						
**Between-variables**													
1.	Baseline mindfulness	2.71	0.50		*0.84*	**0.55**	**−0.47**	**−0.48**	0.22	0.29	**−0.34**	0.12	0.01
2.	Baseline flow experience	3.78	1.09			*0.95*	**−0.39**	−0.14	**0.46**	0.31	**−0.49**	0.10	0.02
3.	Baseline stress	3.09	1.39				*0.90*	0.16	−0.20	**−0.69**	**0.64**	−0.02	−0.01
4.	Trajectory mindfulness	0.03	0.03					–	**0.39**	**−0.35**	0.03	0.07	−0.10
5.	Trajectory flow experience	0.02	0.04						–	−0.06	**−0.34**	0.10	−0.09
6.	Trajectory stress	−0.06	0.02							–	**−0.39**	−0.23	−0.01
7.	Emotional exhaustion	2.67	1.20								*0.89*	−0.03	−0.14
8.	Age	39.98	14.77									–	−0.15
9.	Gender	1.35	0.48										–

*N* = 91. Gender coded as 1 (female) and 2 (male). Significant correlation (*p* < 0.05) are printed in bold. Cronbach’s alpha values are presented in italics on the diagonal.

#### 4.4.2 Analyses of the moderated mediation (between-person level)

For our analyses on the between-person level, we obtained the empirical Bayes estimates for each trajectory from linear mixed models with random intercept and random slope. The slope was allowed to vary among participants to obtain individual growth estimates for each participant. The individual trajectories of mindfulness, flow, and stress were saved as additional variables to allow for a simultaneous assessment of all propositions. This procedure follows previous work by Chen et al. (2011), which was adapted several times to integrate change trajectories in mediation models (e.g., Dust et al., 2021). Treatment was dummy-coded with one equaling participation in the experimental group and zero equaling participation in the control group. We grand-mean centered emotional exhaustion and the trajectory of mindfulness before including their interaction in our model to avoid multicollinearity (Mackinnon et al., 2004).

Firstly, we examined the effects of the MBSR training on the trajectory of flow and stress via the trajectory of mindfulness (model 1). As the next step, we included emotional exhaustion as a moderator (model 2). To test the proposed moderated mediation (see Figure 1), we additionally estimated four conditional indirect effects for higher and lower values of emotional exhaustion (± 1 SD). Initially, we controlled for age, gender, and the individual baseline of mindfulness, stress, and flow experience in both models. Because age and gender did not exert a significant effect on any outcome, we excluded them in the final analysis to avoid biases in parameter estimates due to non-essential covariances (Becker et al., 2016). Table 2 summarizes parameter estimates based on Bayes estimation for model 1 and model 2. Furthermore, we created Johnson-Neyman plots which display the band of significance for the simple slopes across the observed range of the moderator (Bauer and Curran, 2005; Preacher et al., 2006).

**Table 2:** Bayesian mixed models with random intercept and fixed slopes to examine trajectories of mindfulness, flow and stress.

	Model 1				Model 2				Model 3			
	*b*	*SE*	95% CI_low_	95% CI_high_	*b*	*SE*	95% CI_low_	95% CI_high_	*b*	*SE*	95% CI_low_	95% CI_high_
**Predicting trajectory of mindfulness**												
Intercept	**2.71**	0.05	2.62	2.81	**2.81**	0.05	2.72	2.90	**2.81**	0.05	2.72	2.91
Linear change	**0.03**	0.01	0.02	0.04	**1.73**	0.29	1.16	2.29	**1.73**	0.29	1.16	2.29
Quadratic change					−0.36	0.28	−0.89	0.18	−0.35	0.28	−0.89	0.18
Cubic change									−0.25	0.27	−0.77	0.29
**Predicting trajectory of flow experience**												
Intercept	**3.78**	0.12	3.54	4.03	**3.85**	0.12	3.62	4.08	**3.85**	0.12	3.61	4.09
Linear change	**0.02**	0.01	>0.00	0.04	**1.31**	0.64	0.08	2.56	**1.31**	0.65	0.05	2.58
Quadratic change					−0.04	0.63	−1.26	1.20	−0.04	0.62	−1.24	1.21
Cubic change									0.08	0.62	−1.13	1.29
**Predicting trajectory of stress**												
Intercept	**3.02**	0.14	2.76	3.29	**2.80**	0.13	2.55	3.05	**2.80**	0.13	2.56	3.06
Linear change	**−0.06**	0.01	−0.09	−0.04	**−3.75**	0.69	−5.10	−2.40	**−3.77**	0.69	−5.12	−2.42
Quadratic change					1.23	0.67	−0.08	2.52	1.24	0.68	−0.11	2.57
Cubic change									−0.31	0.67	−1.63	0.98

N_within_ = 483, N_between_ = 91. Estimates whose credible interval excludes zero are printed in bold.

## 5 Results

Table 1 summarizes descriptive statistics of the measured variables including means, standard deviations, and Cronbach’s alpha.

### 5.1 Analyses of change trajectories (within-person level)

In support of our first hypotheses, the mixed models with random intercepts and fixed slopes revealed a linear increase in mindfulness and flow experience over the training duration along with a linear decrease in stress (model 1). Neither the quadratic nor cubic slope reached significance for any of the outcomes (models 2 and 3). Please see Table 2 for information about parameter estimates.

### 5.2 Analyses of the moderated mediation (between-person level)

As summarized in Table 3, our results revealed a positive effect of the MBSR training on the trajectory of mindfulness in model 1 (*b* = 0.014, *SE* = 0.005, 95% CI [0.005; 0.023]). Further, in line with our second hypothesis, the present data provided support for the positive effects of the trajectory of mindfulness on the trajectory of flow and negative effects on the trajectory of stress because the corresponding credible intervals exclude zero (flow: *b* = 0.809, *SE* = 0.144, 95% CI [0.530; 1.094]; stress: *b* = −0.251, *SE* = 0.075, 95% CI [−0.396; −0.104]). In order to test the trajectory of mindfulness as a mediator in the relation of the MBSR training to the trajectories of flow and stress, we calculated indirect effects for both outcomes. In line with hypotheses 3a and 3b, we found evidence for a positive indirect effect of the MBSR training on the trajectory of flow (flow: *b* = 0.011, *SE* = 0.004, 95% CI [0.003; 0.020]) and a negative indirect effect of the MBSR training on the trajectory of perceived stress (stress: *b* = −0.004, *SE* = 0.002, 95% CI [−0.007; −0.0004]). Model 1 could explain 30% of the variance of the trajectory of mindfulness, 43% of the variance of the trajectory of flow experience, and 57% of the variance of the trajectory of stress.

**Table 3:** Bayesian estimates for the calculated path model.

	Model 1				Model 2			
	*b*	*SE*	95% CI_low_	95% CI_high_	*b*	*SE*	95% CI_low_	95% CI_high_
**Predicting trajectory of mindfulness**								
Baseline mindfulness	**−0.022**	0.005	−0.031	−0.013	**−0.022**	0.005	−0.031	−0.013
MBSR Intervention	**0.014**	0.005	0.005	0.023	**0.014**	0.005	0.005	0.023
**Predicting trajectory of flow experience**								
Baseline flow experience	**0.013**	0.004	0.006	0.020	**0.012**	0.004	0.005	0.019
Baseline mindfulness	**0.018**	0.008	0.002	0.035	**0.017**	0.008	0.002	0.033
MBSR Intervention	0.001	0.007	−0.012	0.015	0.001	0.006	−0.011	0.014
Trajectory of mindfulness	**0.813**	0.144	0.530	1.094	**0.776**	0.142	0.501	1.053
Emotional exhaustion	−0.003	0.003	−0.009	0.003	−0.003	0.003	−0.009	0.002
Emotional exhaustion × trajectory of mindfulness					**0.237**	0.104	0.030	0.442
**Predicting trajectory of stress**								
Baseline stress	**−0.011**	0.002	−0.014	−0.008	**−0.011**	0.001	−0.014	−0.008
Baseline mindfulness	**−0.009**	0.004	−0.017	−0.002	−0.008	0.004	−0.015	0.000
MBSR intervention	−0.005	0.003	−0.012	0.001	−0.005	0.003	−0.011	0.002
Trajectory of mindfulness	**−0.251**	0.075	−0.396	−0.104	**−0.225**	0.071	−0.364	−0.086
Emotional exhaustion	0.000	0.002	−0.003	0.004	0.001	0.002	−0.003	0.004
Emotional exhaustion × trajectory of mindfulness					**−0.161**	0.051	−0.261	−0.059

*N* = 91. Estimates whose credibility intervals excludes zero are printed in bold.

**Figure 3:**
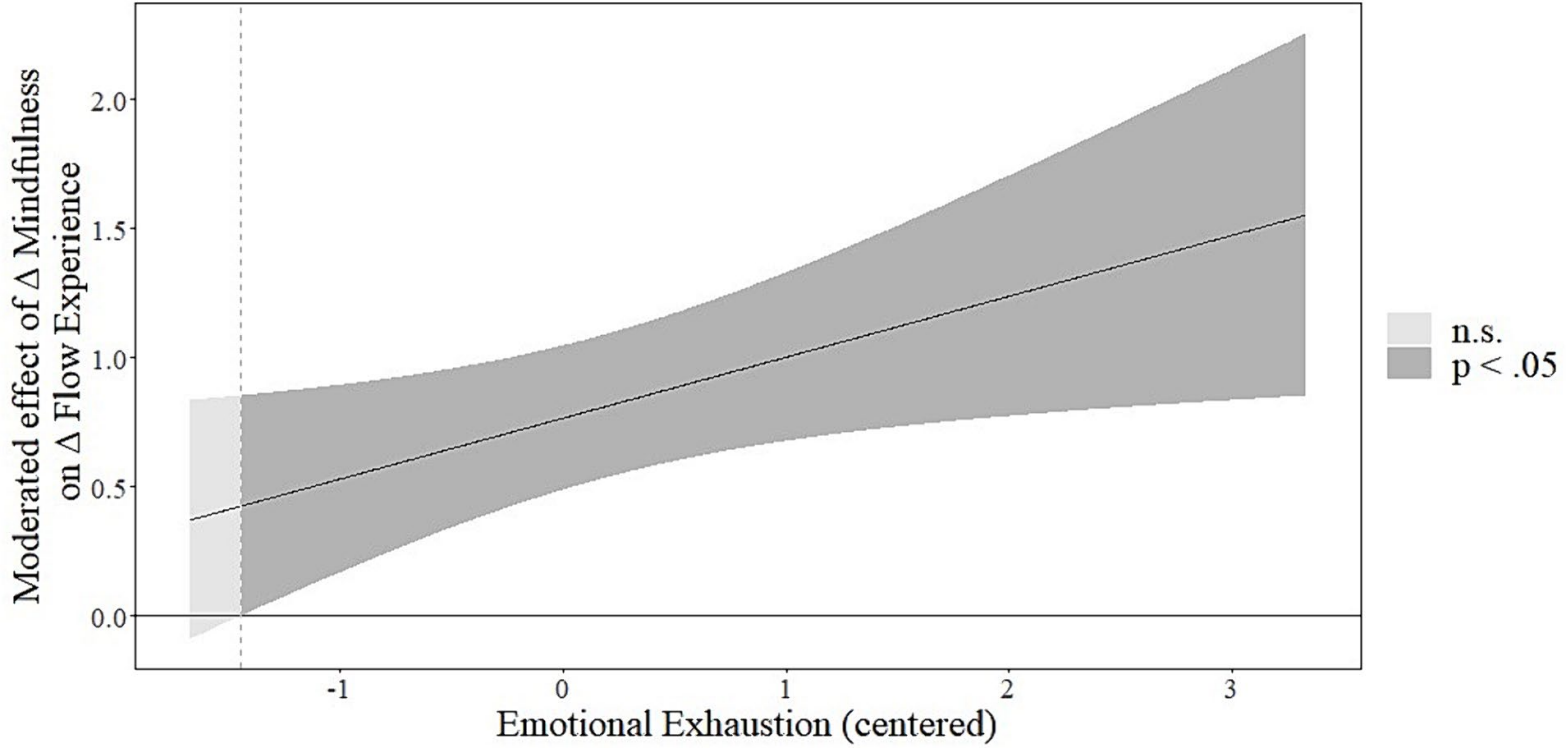
Johnson-Neyman plot showing the interaction between the trajectory of mindfulness and emotional exhaustion on the trajectory of flow experience. Simple slope estimates for the effect of the trajectory of mindfulness and the trajectory of flow experience are displayed on the y-axis depending on emotional exhaustion (x-axis). Emotional exhaustion is plotted only among the centered observed range (min = −1.67, max = 3.33). The moderated effect reaches significance for emotional exhaustion being greater than −1.44.

**Figure 4:**
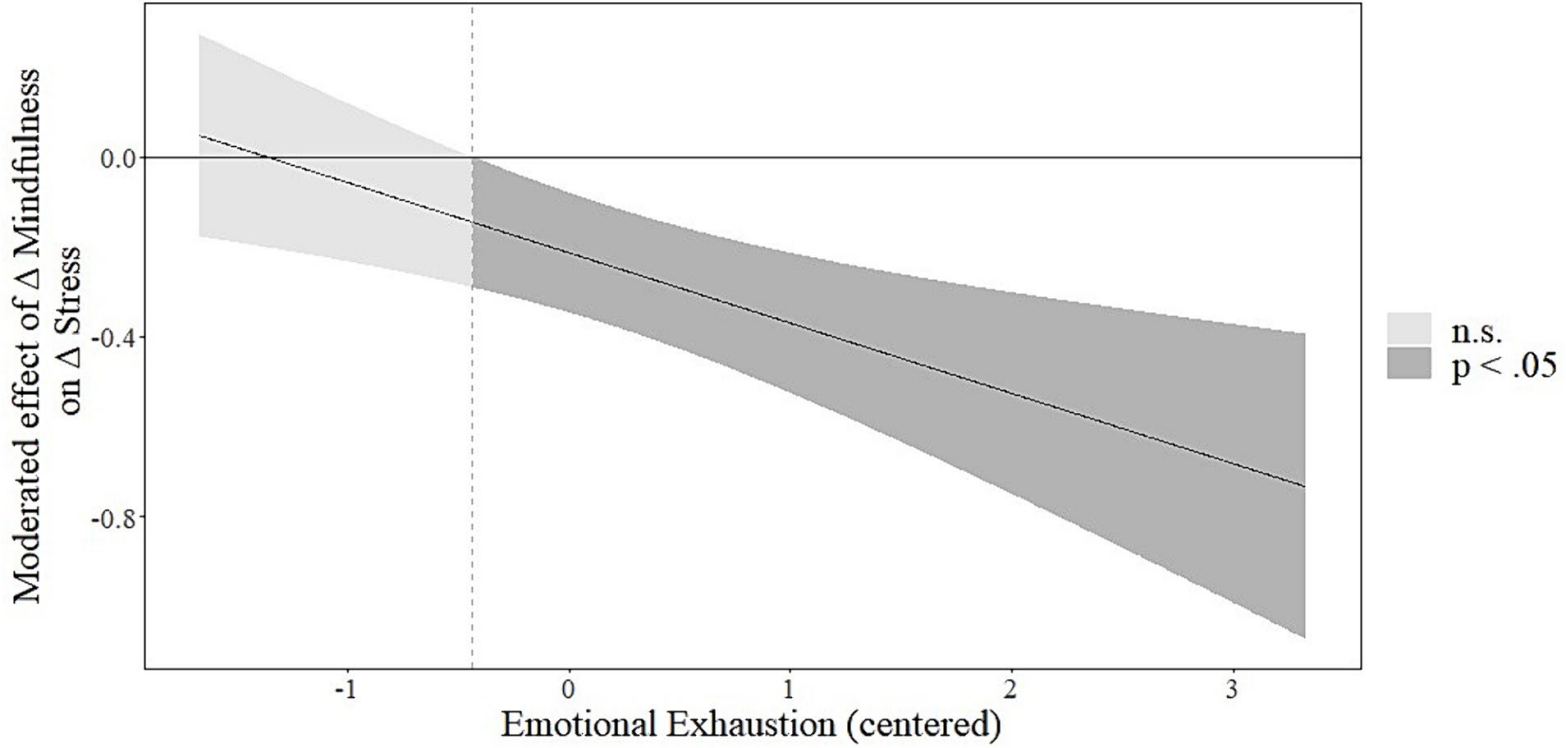
Johnson-Neyman plot showing the interaction between the trajectory of mindfulness and emotional exhaustion on the trajectory of stress. Simple slope estimates for the effect of the trajectory of mindfulness and the trajectory of flow experience are displayed on the y-axis depending on emotional exhaustion (x-axis). Emotional exhaustion is plotted only among the centered observed range (min = −1.67, max = 3.33). The moderated effect reaches significance for emotional exhaustion being greater than −0.46.

As the next step, we tested the moderating effects of emotional exhaustion in model 2. Emotional exhaustion moderated the effects of the trajectory of mindfulness on the trajectory of flow (*b* = 0.237, *SE* = 0.104, 95% CI [0.030; 0.442]) and the trajectory of stress (*b* = −0.161, *SE* = 0.051, 95% CI [−0.261; −0.059]), supporting hypotheses 4a and 4b. The Johnson-Neyman plots (depicted in Figures 3, 4) show that emotional exhaustion strengthens the positive and negative relationships between the trajectory of mindfulness and, respectively, the trajectory of flow and the trajectory of stress. Further, we calculated the conditional indirect effects of the MBSR training on the trajectories of flow and stress via the trajectory of mindfulness for higher and lower values of emotional exhaustion (± 1 SD). The results reveal a positive conditional indirect effect on the trajectory of flow for individuals with high emotional exhaustion (*b* = 0.015, *SE* = 0.006, 95% CI [0.004; 0.026]), but not for individuals with low emotional exhaustion (*b* = 0.007, *SE* = 0.004, 95% CI [−0.0005; 0.014]). Similarly, we found a negative conditional indirect effect on the trajectory of stress for individuals with high emotional exhaustion (*b* = −0.006, *SE* = 0.002, 95% CI [−0.011; −0.001]), but not for individuals with low emotional exhaustion (*b* = −0.0005, *SE* = 0.001, 95% CI [−0.003; 0.002]). Hence, our results support hypotheses 5a and 5b. Model 2 could explain 30% of the variance of the trajectory of mindfulness, 44% of the variance of the trajectory of flow experience (∆R^2^ = 0.02), and 61% of the variance of the trajectory of stress (∆R^2^ = 0.04).

## 6 Discussion

Our study explored the temporal changes in mindfulness, stress, and flow experience during the MBSR program as well as their relations. Building on previous research, we found a consistent linear increase in mindfulness and a linear decrease in stress over the training duration. Additionally, based on the TSF (Peifer and Tan, 2021), our findings supported the notion that the MBSR training can promote optimal functioning, as indicated by a linear increase in flow experience. Moreover, we investigated the moderating role of emotional exhaustion and revealed that individuals with high emotional exhaustion benefited more from the effects of mindfulness on flow experience and stress reduction.

Whereas some interventions have been implemented in sports to foster flow experience (e.g., Koehn et al., 2014; Scott-Hamilton et al., 2016), only a few have been developed for the workplace (for an exception see Bartzik et al., 2021). Our findings support the idea that mindfulness and related interventions, such as the MBSR training, have the capacity to foster flow, despite certain dimensions appearing incompatible. While flow and mindfulness have been regarded as conceptually divergent, with mindfulness involving broad attentional awareness and self-awareness while flow entails a narrow focus without self-awareness (Sheldon et al., 2015), our study supports the proposition that the development of mindfulness over time can facilitate flow. By cultivating a clearer perception of demanding situations and efficient allocation of cognitive resources, individuals can gradually shift their interpretation of these situations as positive challenges rather than stressors. This notion from the TSF (Peifer and Tan, 2021) is also supported by previous research indicating the positive impact of mindfulness on the reappraisal of situations (Garland et al., 2015, 2017). By revealing a positive relationship between temporal changes in mindfulness and flow during the MBSR training, our study bridges the gap between the incompatibility of certain dimensions of mindfulness and flow and the potential of mindfulness to enhance flow. Taking a longitudinal perspective in investigating the effects of mindfulness on flow, we extend previous research that primarily relied on pre- and post-measurements of mindfulness interventions and enhance our understanding of the dynamic relationship between these constructs, which can inform the development of more effective mindfulness-based interventions to promote optimal functioning at work. In future research, it is important to explore and compare the effects of different mindfulness-based interventions on flow, considering factors such as the duration of interventions, specific exercises employed (e.g., breathing exercises, body scans, meditation), and the target audience (e.g., employees, leaders), to further enhance our knowledge in this area.

Furthermore, our study adds to the current literature on the effects of mindfulness interventions by investigating the shape of the trajectories of mindfulness, stress, and flow over time. Previous research has largely overlooked the diverse change patterns and their relationships within mindfulness interventions. By examining the shape of trajectories of these variables, we provide a deeper understanding of how the MBSR program unfolds and its effects on individuals in the workplace. Our results indicate a positive linear increase in mindfulness and a linear decrease in stress over the training duration, aligning with previous studies that have also observed a gradual increase in these variables during MBSR training (Baer et al., 2012; Snippe et al., 2017) and other mindfulness-based interventions over several weeks (Garland et al., 2017; Andreotti et al., 2018). Contrary to results from Baer et al. (2012), who revealed a reduction in stress only starting in the third week, and the findings from a qualitative analysis by Isbel et al. (2020), who revealed greater effort toward the beginning and greater benefits toward the end of a long-term intervention, a linear change implies a continuous growth of mindfulness and reduction of stress, independently of the current time point. Despite the aim of most mindfulness-based interventions to increase optimized functioning (Kabat-Zinn, 2003; Good et al., 2016), surprisingly, the temporal changes in flow experience during the MBSR program have not been extensively examined. Our study fills this gap by revealing a linear increase in flow experience, expanding our understanding of the effects of mindfulness interventions on individuals’ overall wellbeing. In light of previous research showing curvilinear trajectories in other wellbeing indicators such as rumination and heart rate variability (Andreotti et al., 2018; Krick et al., 2021), future studies shall continue to explore the trajectories of various indicators of wellbeing separately. Accordingly, future studies may also assess how other indicators of wellbeing and optimal functioning that have been investigated as outcomes of the MBSR training, such as emotional regulation, quality of life, or physical health (Eberth and Sedlmeier, 2012; Vibe et al., 2017), develop during the training duration.

Lastly, as predicted by our fourth hypothesis, emotional exhaustion amplified the positive effect of the trajectory of mindfulness on the trajectory of flow experience as well as the negative effect on the trajectory of stress. Especially in demanding sectors, such as health care or education, where emotional exhaustion tends to be concerningly high (Bridgeman et al., 2018; Park and Shin, 2020), a mindfulness-based intervention can be helpful to reduce stress and enable the experience of flow, which has been associated with higher wellbeing and performance (Peifer and Wolters, 2021). These results are particularly important in light of recent research that revealed potential negative effects of mindfulness and meditation such as increased stress, anxiety, or negative affect under certain circumstances (e.g., Britton, 2019; Farias et al., 2020; Britton et al., 2021). By examining the impact of the MBSR training on the wellbeing of chronically depleted individuals, we ensure that those who may be vulnerable to side effects can still benefit from the intervention, leading to enhanced wellbeing rather than diminished outcomes. Furthermore, in the long term, higher levels of mindfulness may also reduce emotional exhaustion (Hülsheger et al., 2013; Good et al., 2016). In line with this, in a randomized controlled trial, Verweij et al. (2018) could reveal a negative effect of the MBSR training on emotional exhaustion for individuals with high baseline levels of emotional exhaustion. These results conform with our proposition that the MBSR training is more beneficial for chronically depleted individuals and additionally imply circular effects (i.e., the intervention reduces emotional exhaustion which in turn reduces the beneficial effects of the intervention on emotional exhaustion). Further, emotional exhaustion and flow experience show a negative relationship (Aust et al., 2022), so that the training might be even more effective to increase optimal functioning for depleted individuals. Future studies that investigate the effects of mindfulness-based interventions on indicators of wellbeing and performance should take these results into account and test for possible baseline interactions as well as circular effects.

### 6.1 Limitations

In the following, we discuss several limitations of the present research. First, since our participants were not randomly allocated to the experimental and control groups, a selection bias might lead to systematic differences between the two groups such as different interests in participation in a mindfulness-based stress reduction training. Even though both groups did not statistically differ in their age, the initial level of mindfulness, flow, or emotional exhaustion and we controlled for age, gender as well as possible baseline effects, future studies should consider an experimental design with randomization and an active control group. Second, all data has been collected retrospectively each week via self-report. Thus, the common method bias and memory biases could lead to a potential measurement error (Podsakoff et al., 2003). Further studies could additionally assess physiological correlates over longer time periods of flow experience and stress, such as heart rate variability or cortisol levels, to overcome this limitation (e.g., Peifer and Tan, 2021). Further, neuroplasticity represents an important physiological indicator of mindfulness that is not directly linked to stress responses and might be integrated into further research as well (Good et al., 2016). Third, we measured mindfulness, flow experience, and stress simultaneously over the duration of 8 weeks. Even though the MBSR training allows us to manipulate mindfulness, we cannot ensure a causal effect of mindfulness on flow experience and stress with our design. A bi-directional relation should be investigated in future studies. Fourth, due to the longitudinal design of our study, we faced a considerable dropout during the intervention, with only 21 and 26 participants in the experimental and control group, respectively, during the last week. The relatively small sample size at the end of the study period reduces statistical power, which decreases the chances to reveal small effects, but also increases the risk of a sample bias. Fifth, because of this longitudinal design, we measured mindfulness with the CAMS-R questionnaire as a relatively short questionnaire (Feldman et al., 2007). However, the questionnaire does not allow a separate investigation of different sub-facets. Because different facets such as acceptance or attentional control might have a different influence on flow experience and stress, future research should investigate those separately. Despite the limitations discussed, the current study provides an important starting point to better understand how and for whom long-term mindfulness interventions foster flow experience and reduce stress over time.

## 7 Conclusion

In conclusion, we were able to shed light on how an MBSR intervention enhances wellbeing in the work context and for whom mindfulness is most beneficial. Based on the TSF, we provide a profound theoretical explanation of how increases in mindfulness are associated with increases in flow and decreases in stress during the MBSR training. In doing so, this research expands the application of mindfulness in the workplace by examining flow experience as an indicator of optimal psychological functioning. Additionally, we demonstrate that the program is particularly beneficial for individuals with high emotional exhaustion. These findings highlight the potential of the MBSR intervention to support the wellbeing and mental health of depleted employees.

## Data availability statement

The original contributions presented in the study are included in the article/Supplementary material, further inquiries can be directed to the corresponding authors.

## Ethics statement

The studies involving humans were approved by the ethics committee of the University of Wuppertal. The studies were conducted in accordance with the local legislation and institutional requirements. The participants provided their written informed consent to participate in this study.

## Author contributions

CH: Writing – review & editing, Writing – original draft, Methodology, Investigation, Formal analysis, Conceptualization. FE: Writing – review & editing, Methodology, Investigation, Conceptualization. CP: Writing – review & editing, Supervision, Methodology, Conceptualization. SD: Writing – review & editing, Supervision, Methodology, Formal analysis, Conceptualization.

## Footnotes

^1^Results show similar patterns and significance when all participants are included.
