# Supplementary material for: Trajectories of mindfulness, flow experience, and stress during an online-based MBSR program: the moderating role of emotional exhaustion
Source: Front Psychol. 2024 Jul 15;15:1385372. doi: 10.3389/fpsyg.2024.1385372 (PMC11285067; doi:10.3389/fpsyg.2024.1385372)
Supplement: Supplementary file 2 [file Table_2.DOCX]

library(openxlsx)

library(misty)

library(plyr)

library(dplyr)

library(tidyr)

library(brms)

library(rstan)

library(ggmcmc)

library(ggplot2)

library(mcmcplots)

library(performance)

library(psych)

library(blavaan)

library(lsr)

###### Read and prepare data #######

Dat <- read.xlsx("Data_Trajectories_MBSR.xlsx")

## Overview

table(Dat$Gruppe, Dat$Fragebogen)

Dat$Fragebogen <- as.numeric(as.factor(Dat$Fragebogen)) # 1 = Baseline Measure, 9 = Week 8

Dat$Fragebogen <- Dat$Fragebogen - 1 # 0 = Baseline Measure, 8 = Week 8

## Listwise deletion

Dat2 <- Dat[-c(which(is.na(Dat$FH_Mean)),

which(is.na(Dat$IR_Mean)),

which(is.na(Dat$CA_Mean)),

which(is.na(Dat$BO_EE_Mean))

),]

## Save baseline Measures as additional level 2 variable

Dat2$T0_FH <- Dat2$FH_Mean

Dat2$T0_IR <- Dat2$IR_Mean

Dat2$T0_CA <- Dat2$CA_Mean

Dat2 <- Dat2 %>% mutate_at(vars("T0_FH", "T0_IR", "T0_CA"), ~ifelse(Fragebogen != "0", NA, .))

Dat2 <- Dat2 %>%

group_by(SERIAL) %>%

fill(c("T0_FH", "T0_IR", "T0_CA"), .direction="downup")

## Dataset with one row per person (for preliminary analyses)

d_between <- Dat2[Dat2$Fragebogen == 0,]

any(duplicated(d_between$SERIAL))

## Participant numbers (Figure 1)

table(Dat2$Gruppe, Dat2$Fragebogen)

#### Preliminary analyses: sample characteristics and response rate #####

# Differences in sample characteristics

d_between$Gruppe <- as.numeric(as.factor(d_between$Gruppe)) # 1 = KG, 2 = EG

d_between$Gruppe <- 2 - d_between$Gruppe # 0 = KG, 1 = EG

summary(as.factor(d_between$Gruppe))

# Response rate

count <- as.data.frame(count(Dat2, "SERIAL"))

d_between <- full_join(d_between, count)

summary(as.factor(d_between$n))

t.test(d_between[d_between$Gruppe==1, "DD0101"], d_between[d_between$Gruppe==0, "DD0101"]) ## significant

t.test(d_between[d_between$Gruppe==1, "DD02"], d_between[d_between$Gruppe==0, "DD02"])

t.test(d_between[d_between$Gruppe==1, "BO_EE_Mean"], d_between[d_between$Gruppe==0, "BO_EE_Mean"])

t.test(d_between[d_between$Gruppe==1, "T0_CA"], d_between[d_between$Gruppe==0, "T0_CA"])

t.test(d_between[d_between$Gruppe==1, "T0_IR"], d_between[d_between$Gruppe==0, "T0_IR"]) ## significant

t.test(d_between[d_between$Gruppe==1, "T0_FH"], d_between[d_between$Gruppe==0, "T0_FH"])

t.test(d_between[d_between$Gruppe==1, "n"], d_between[d_between$Gruppe==0, "n"])

describeBy(d_between, group="Gruppe")

###### Multi-level CFA #####

library (lavaan)

model <- '

level: 1

F_CA =~ CA01 + CA02 + CA03 + CA04 + CA05 + CA06 + CA07 + CA08 + CA09 + CA10 + CA11 + CA12

F_IR1 =~ IR01 + IR02 + IR04

F_IR2 =~ IR03 + IR05 + IR06 + IR07 + IR08

F_IR =~ F_IR1 + F_IR2

F_FH1 =~ FH01 + FH02 + FH04

F_FH2 =~ FH05 + FH07 + FH08

F_FH3 =~ FH03 + FH06 + FH09 + FH10

F_FH =~ F_FH1 + F_FH2+ F_FH3

level: 2

F_EE =~ BO01 + BO02 + BO03 + BO04 + BO05 + BO06'

fit <- sem(model = model, data = Dat2, cluster = "SERIAL")

summary(fit, fit.measures = TRUE)

model2 <- '

level: 1

F_CA =~ CA01 + CA02 + CA03 + CA04 + CA05 + CA06 + CA07 + CA08 + CA09 + CA10 + CA11 + CA12

F_IR1 =~ IR01 + IR02 + IR04

F_IR2 =~ IR03 + IR05 + IR06 + IR07 + IR08

F_FH1 =~ FH01 + FH02 + FH04

F_FH2 =~ FH05 + FH07 + FH08

F_FH3 =~ FH03 + FH06 + FH09 + FH10

F_FHIR =~ F_FH1 + F_FH2+ F_FH3 + F_IR1 + F_IR2

level: 2

F_EE =~ BO01 + BO02 + BO03 + BO04 + BO05 + BO06'

fit2 <- sem(model = model2, data = Dat2, cluster = "SERIAL")

summary(fit2, fit.measures = TRUE)

model3 <- '

level: 1

F_CA =~ CA01 + CA02 + CA03 + CA04 + CA05 + CA06 + CA07 + CA08 + CA09 + CA10 + CA11 + CA12

F_IR1 =~ IR01 + IR02 + IR04

F_IR2 =~ IR03 + IR05 + IR06 + IR07 + IR08

F_IR =~ F_IR1 + F_IR2

F_FH1 =~ FH01 + FH02 + FH04

F_FH2 =~ FH05 + FH07 + FH08

F_FH3 =~ FH03 + FH06 + FH09 + FH10

F_FHCA =~ F_FH1 + F_FH2+ F_FH3 + F_CA

level: 2

F_EE =~ BO01 + BO02 + BO03 + BO04 + BO05 + BO06'

fit3 <- sem(model = model3, data = Dat2, cluster = "SERIAL")

summary(fit3, fit.measures = TRUE)

model4 <- '

level: 1

F_CA =~ CA01 + CA02 + CA03 + CA04 + CA05 + CA06 + CA07 + CA08 + CA09 + CA10 + CA11 + CA12

F_IR1 =~ IR01 + IR02 + IR04

F_IR2 =~ IR03 + IR05 + IR06 + IR07 + IR08

F_FH1 =~ FH01 + FH02 + FH04

F_FH2 =~ FH05 + FH07 + FH08

F_FH3 =~ FH03 + FH06 + FH09 + FH10

F_FH =~ F_FH1 + F_FH2+ F_FH3

F_IRCA =~ F_IR1 + F_IR2 + F_CA

level: 2

F_EE =~ BO01 + BO02 + BO03 + BO04 + BO05 + BO06'

fit4 <- sem(model = model4, data = Dat2, cluster = "SERIAL")

summary(fit4, fit.measures = TRUE)

###### Test variance within and among participants #####

## Get ICC based on random-intercept model

icc1 <- brm(CA_Mean ~1 + (1|SERIAL), Dat2, iter = 6000, cores=8)

icc2 <- brm(FH_Mean ~1 + (1|SERIAL), Dat2, iter = 6000, cores=8)

icc3 <- brm(IR_Mean ~1 + (1|SERIAL), Dat2, iter = 6000, cores=8)

summary(icc1)

summary(icc2)

summary(icc3)

icc(icc1)

icc(icc2)

icc(icc3)

###### Analyze trajectories #######

# compare linear, quadratic & cubic slopes (random intercept, fixed slopes)

# linear slope

lm1 <- brm(CA_Mean ~ Fragebogen + 1 + (1|SERIAL), Dat2, iter = 6000, cores=8) # linear slope

lm2 <- brm(FH_Mean ~ Fragebogen + 1 + (1|SERIAL), Dat2, iter = 6000, cores=8) # linear slope

lm3 <- brm(IR_Mean ~ Fragebogen + 1 + (1|SERIAL), Dat2, iter = 6000, cores=8) # linear slope

print(summary (lm1), digits=3) # linear slope

print(summary (lm2), digits=3) # linear slope

print(summary (lm3), digits=3) # linear slope

# quadratic slope

qm1 <- brm(CA_Mean ~ poly(Fragebogen,2) + 1 + (1|SERIAL), Dat2, iter = 6000, cores=8) # quadratic slope

qm2 <- brm(FH_Mean ~ poly(Fragebogen,2) + 1 + (1|SERIAL), Dat2, iter = 6000, cores=8) # quadratic slope

qm3 <- brm(IR_Mean ~ poly(Fragebogen,2) + 1 + (1|SERIAL), Dat2, iter = 6000, cores=8) # quadratic slope

print(summary (qm1), digits=3) # quadratic slope

print(summary (qm2), digits=3) # quadratic slope

print(summary (qm3), digits=3) # quadratic slope

# cubic slope

cm1 <- brm(CA_Mean ~ poly(Fragebogen,3) + 1 + (1|SERIAL), Dat2, iter = 6000, cores=8) # cubic slope

cm2 <- brm(FH_Mean ~ poly(Fragebogen,3) + 1 + (1|SERIAL), Dat2, iter = 6000, cores=8) # cubic slope

cm3 <- brm(IR_Mean ~ poly(Fragebogen,3) + 1 + (1|SERIAL), Dat2, iter = 6000, cores=8) # cubic slope

print(summary (cm1), digits=3) # cubic slope

print(summary (cm2), digits=3) # cubic slope

print(summary (cm3), digits=3) # cubic slope

###### Adding individual linear trajectories as manifest variables to the data set #######

## Calculate random intercept, random slope models (linear slope) to get individual growth estimates

fm1_r <- brm(CA_Mean ~ Fragebogen + 1 + (Fragebogen|SERIAL), Dat2, iter = 6000, cores = 8)

fm2_r <- brm(FH_Mean ~ Fragebogen + 1 + (Fragebogen|SERIAL), Dat2, iter = 6000, cores = 8)

fm3_r <- brm(IR_Mean ~ Fragebogen + 1 + (Fragebogen|SERIAL), Dat2, control = list( adapt_delta = .999), iter = 6000, cores = 8) # increases adapt_delta because of divergent interations

Dat3 <- Dat2

# Intercept & Slope Mindfulness

summary(fm1_r)

CA_Slope <- as.data.frame(coef(fm1_r)$SERIAL)

CA_Slope$SERIAL <- row.names(CA_Slope)

names(CA_Slope)

CA_Slope <- CA_Slope[, c("Estimate.Intercept", "Estimate.Fragebogen", "SERIAL" )]

names(CA_Slope)

colnames(CA_Slope)<- c( "CA_Int", "CA_Slope", "SERIAL")

CA_Slope$SERIAL <- as.numeric(CA_Slope$SERIAL)

Dat3<- full_join(Dat3, CA_Slope)

# Intercept & Slope Flow

summary(fm2_r)

FH_Slope <- as.data.frame(coef(fm2_r)$SERIAL)

FH_Slope$SERIAL <- row.names(FH_Slope)

FH_Slope <- FH_Slope[, c("Estimate.Intercept", "Estimate.Fragebogen", "SERIAL" )]

names(FH_Slope)

colnames(FH_Slope)<- c( "FH_Int", "FH_Slope", "SERIAL")

FH_Slope$SERIAL <- as.numeric(FH_Slope$SERIAL)

Dat3<- full_join(Dat3, FH_Slope)

# Intercept & Slope Stress

summary(fm3_r)

IR_Slope <- as.data.frame(coef(fm3_r)$SERIAL)

IR_Slope$SERIAL <- row.names(IR_Slope)

IR_Slope <- IR_Slope[, c("Estimate.Intercept", "Estimate.Fragebogen", "SERIAL" )]

names(IR_Slope)

colnames(IR_Slope)<- c( "IR_Int", "IR_Slope", "SERIAL")

IR_Slope$SERIAL <- as.numeric(IR_Slope$SERIAL)

Dat3<- full_join(Dat3, IR_Slope)

###### Descriptive Statistics ######

Dat3 = ddply(Dat3, .(SERIAL), mutate, CA_Mean_GP = mean(CA_Mean, na.rm=T)) #Group Mean

Dat3 = ddply(Dat3, .(SERIAL), mutate, FH_Mean_GP = mean(FH_Mean, na.rm=T)) #Group Mean

Dat3 = ddply(Dat3, .(SERIAL), mutate, IR_Mean_GP = mean(IR_Mean, na.rm=T)) #Group Mean

Dat3 = ddply(Dat3, .(SERIAL), mutate, CA_Mean_sd = sd(CA_Mean, na.rm=T)) #SD within

Dat3 = ddply(Dat3, .(SERIAL), mutate, FH_Mean_sd = sd(FH_Mean, na.rm=T)) #SD within

Dat3 = ddply(Dat3, .(SERIAL), mutate, IR_Mean_sd = sd(IR_Mean, na.rm=T)) #SD within

d_between <- Dat3[Dat3$Fragebogen == 0,]

any(duplicated(d_between$SERIAL))

names(d_between)

## Descriptives

describe(d_between[,c(53,51,52,55,57,59,50,4,5,60:62 )])

describe(d_between[,63:65]) # SD within

## Cronbachs Alpha

Dat3 <- ungroup(Dat3)

# CA

for (x in 0:8) {

filename <- paste("T", x, sep="")

T<-alpha(select(Dat3[Dat3$Fragebogen==x,], num_range("CA", 1:12, width =2)), check.keys = T) [1] ### save raw alpha

T<-unlist(T)[1] ### save raw alpha

assign(filename, T)

}

CA_min <- min(T0, T1, T2, T3, T4, T5, T6, T7, T8)

CA_max <- max(T0, T1, T2, T3, T4, T5, T6, T7, T8)

CA_T0 <- T0

rm(T, T0, T1, T2, T3, T4, T5, T6, T7, T8)

# FH

for (x in 0:8) {

filename <- paste("T", x, sep="")

T<-alpha(select(Dat3[Dat3$Fragebogen==x,], num_range("FH", 1:10, width =2)), check.keys = T) [1] ### save raw alpha

T<-unlist(T)[1] ### save raw alpha

assign(filename, T)

}

FH_min <- min(T0, T1, T2, T3, T4, T5, T6, T7, T8)

FH_max <- max(T0, T1, T2, T3, T4, T5, T6, T7, T8)

FH_T0 <- T0

rm(T, T0, T1, T2, T3, T4, T5, T6, T7, T8)

# IR

for (x in 0:8) {

filename <- paste("T", x, sep="")

T<-alpha(select(Dat3[Dat3$Fragebogen==x,], num_range("IR", 1:8, width =2)), check.keys = T) [1] ### save raw alpha

T<-unlist(T)[1] ### save raw alpha

assign(filename, T)

}

IR_min <- min(T0, T1, T2, T3, T4, T5, T6, T7, T8)

IR_max <- max(T0, T1, T2, T3, T4, T5, T6, T7, T8)

IR_T0 <- T0

rm(T, T0, T1, T2, T3, T4, T5, T6, T7, T8)

# BO

alpha(select(Dat3[Dat3$Fragebogen==0,], num_range("BO", 1:6, width =2)), check.keys = T)

## Correlations

corr.test(d_between[,c(53,51,52,55,57,59,50,4,5)])

names(Dat3)

corr.test(Dat3[,c(48,49,47)])

## Demographics

summary(as.factor(d_between$DD02))

summary(as.factor(d_between$DD02))/91

describe(d_between$DD0101)

summary(as.factor(d_between$DD03))/91

# 1 = working (Berufstätig)

# 2 = education at a company (in Ausbildung)

# 3 = student (Student)

# 4 = pupil (Schüler)

# 5 = on job search (Arbeitssuchend)

# 6 = household, damily, private care taling (in Haushalt/ Familie/ privater Pflege tätig)

# 7 = retired (Rentner/ Pensionär)

# 8 = voluntary work (ehrenamtlich tätig)

summary(as.factor(d_between$DD07))/91

# 1 = Highschool certificate (Abitur)

# 2 = Highschool certificate - limited university entree (Fachabitur)

# 3 = Middle School (Mittlere Reife)

# 4 = Middle School - lower level (Hauptschulabschluss)

# 5 = no school certificate (kein Schulabschluss)

# 6 = Bachelor

# 7 = Master

# 8 = Diploma (Diplom)

# 9 = PhD (Doktor)

# 10 = Professor

# 11 = others (Sonstiges)

###### Examination of between person effects #####

### Center & recode

d_between$CA_Slope_c <- scale(d_between$CA_Slope, scale = F)

d_between$BO_EE_Mean_c<- scale(d_between$BO_EE_Mean, scale = F)

d_between$Gruppe <- as.numeric(as.factor(d_between$Gruppe))

d_between$Gruppe <- 2 - d_between$Gruppe # 0 = KG, 1 = EG

###### Path model

describe(d_between$BO_EE_Mean)

describe(d_between$BO_EE_Mean_c)

### Model 1 with age & gender

model1<-'

# direct effects

FH_Slope ~ b1*CA_Slope_c + c1*Gruppe + d1*BO_EE_Mean_c + k1*T0_CA + k2*T0_FH + h1*DD0101 + h2*DD02

IR_Slope ~ b2*CA_Slope_c + c2*Gruppe + d2*BO_EE_Mean_c + k3*T0_CA + k4*T0_IR + h3*DD0101 + h4*DD02

CA_Slope_c~ a*Gruppe + k5*T0_CA + h5*DD0101 + h6*DD02

# variances

FH_Slope ~~ FH_Slope

IR_Slope ~~ IR_Slope

# indirect effects

ind1 := a*b1

ind2 := a*b2

total:= ind1 + ind2 + c1 + c2

'

library(stan)

library(blavaan) # to make sure it doesn't interfere with lavaan

fit_model1 <- bsem(model1, data = d_between, bcontrol=list(cores=8, control=list(max_treedepth = 15)), burnin = 1000, sample = 6000)

summary(fit_model1, rsquare=T)

### Model 2 with age and gender

model2<-'

# direct effects

FH_Slope ~ b1*CA_Slope_c + c1*Gruppe + d1*BO_EE_Mean_c + m1*BO_EE_Mean_c:CA_Slope_c + k1*T0_CA + k2*T0_FH + h1*DD0101 + h2*DD02

IR_Slope ~ b2*CA_Slope_c + c2*Gruppe + d2*BO_EE_Mean_c + m2*BO_EE_Mean_c:CA_Slope_c + k3*T0_CA + k4*T0_IR + h3*DD0101 + h4*DD02

CA_Slope_c ~ a*Gruppe + k5*T0_CA + h5*DD0101 + h6*DD02

# Conditional effects b path

b1_h := b1 + (1.2*m1)

b1_l := b1 - (1.2*m1)

b2_h := b2 + (1.2*m2)

b2_l := b2 - (1.2*m2)

#Variances

FH_Slope ~~ FH_Slope

IR_Slope ~~ IR_Slope

# indirect effects

ind1_h := a*b1_h

ind1_l := a*b1_l

ind2_h := a*b2_h

ind2_l := a*b2_l

ind1 := a*b1

ind2 := a*b2

total:= ind1 + ind2 + c1 + c2

'

library(blavaan) # to make sure it doesn't interfere with lavaan

fit_model2 <- bsem(model2, data = d_between, bcontrol=list(cores=8, control=list(max_treedepth = 15)), burnin = 1000, sample = 6000)

summary(fit_model2, rsquare=T)

### Model 1 without age & gender

model1_final<-'

# direct effects

FH_Slope ~ b1*CA_Slope_c + c1*Gruppe + d1*BO_EE_Mean_c + k1*T0_CA + k2*T0_FH

IR_Slope ~ b2*CA_Slope_c + c2*Gruppe + d2*BO_EE_Mean_c + k3*T0_CA + k4*T0_IR

CA_Slope_c~ a*Gruppe + k5*T0_CA

# variances

FH_Slope ~~ FH_Slope

IR_Slope ~~ IR_Slope

# indirect effects

ind1 := a*b1

ind2 := a*b2

total:= ind1 + ind2 + c1 + c2

'

library(blavaan) # to make sure it doesn't interfere with lavaan

fit_model1_final <- bsem(model1_final, data = d_between, bcontrol=list(cores=8), burnin = 1000, sample = 6000)

summary(fit_model1_final, rsquare=T, nd =5)

### Model 2 without age and gender

model2_final<-'

# direct effects

FH_Slope ~ b1*CA_Slope_c + c1*Gruppe + d1*BO_EE_Mean_c + m1*BO_EE_Mean_c:CA_Slope_c + k1*T0_CA + k2*T0_FH

IR_Slope ~ b2*CA_Slope_c + c2*Gruppe + d2*BO_EE_Mean_c + m2*BO_EE_Mean_c:CA_Slope_c + k3*T0_CA + k4*T0_IR

CA_Slope_c ~ a*Gruppe + k5*T0_CA

# Conditional effects b path

b1_h := b1 + (1.2*m1)

b1_l := b1 - (1.2*m1)

b2_h := b2 + (1.2*m2)

b2_l := b2 - (1.2*m2)

#Variances

FH_Slope ~~ FH_Slope

IR_Slope ~~ IR_Slope

# indirect effects

ind1_h := a*b1_h

ind1_l := a*b1_l

ind2_h := a*b2_h

ind2_l := a*b2_l

ind1 := a*b1

ind2 := a*b2

total:= ind1 + ind2 + c1 + c2

'

library(blavaan) # to make sure it doesn't interfere with lavaan

fit_model2_final <- bsem(model2_final, data = d_between, bcontrol=list(cores=8), burnin = 1000, sample = 6000)

summary(fit_model2_final, rsquare=T, nd =5)

#### johnson.neyman plots

library(interactions)

library(ggplot2)

library(extrafont)

loadfonts(device = "win", quiet = T)

#fonts()

### write function to create the plots (based on the function "johnson_neyman" from the interactions package)

johnson_neyman_blavaan <- function(model, data, pred, pred2, modx, modx2, intterm, intterm2, outcome,

alpha = 0.05,

plot = TRUE, control.fdr = FALSE,

line.thickness = 0.5,

digits = getOption("jtools-digits", 2),

sig.color = "#00BFC4",

insig.color = "#F8766D", mod.range = NULL,

title = "Johnson-Neyman plot") {

out <- list()

out <- structure(out, pred = pred, modx = modx, alpha = alpha,

plot = plot, digits = digits, control.fdr = control.fdr)

# Getting the range of the moderator

modrange <- range(data[,modx])

modrangeo <- range(data[,modx]) # for use later

modsd <- sd( data[,modx]) # let's expand outside observed range

if (is.null(mod.range)) {

modrange[1] <- modrange[1] - modsd

modrange[2] <- modrange[2] + modsd

} else {modrange <- mod.range}

if (modrange[1] >= modrangeo[1] & modrange[2] <= modrangeo[2]) {

no_range_line <- TRUE

} else {no_range_line <- FALSE}

alpha <- alpha / 2

# Large Sample Approximation for Critical test Statistic

tcrit <- qt(alpha, df = Inf)

# Reverse the sign since it gives negative at these low vals

tcrit <- abs(tcrit)

# Construct constituent terms to calculate the subsequent quadratic a,b,c

# Get vcov

vmat <- vcov(model)

# Variance of interaction term (gamma_3)

covy3 <- vmat[intterm2,intterm2]

# Variance of predictor term (gamma_1)

covy1 <- vmat[pred2,pred2]

# Covariance of predictor and interaction terms (gamma_1 by gamma_3)

covy1y3 <- vmat[intterm2,pred2]

# Actual interaction coefficient (gamma_3)

y3 <- blavInspect(model, "est")$beta[outcome, intterm]

# Actual predictor coefficient (gamma_1)

y1 <- blavInspect(model, "est")$beta[outcome, pred]

# Now we use this info to construct a quadratic equation

a <- tcrit^2 * covy3 - y3^2

b <- 2 * (tcrit^2 * covy1y3 - y1*y3)

c <- tcrit^2 * covy1 - y1^2

# Now we define a function to test for number of real solutions to it

## The discriminant can tell you how many there will be

discriminant <- function(a,b,c) {

disc <- b^2 - 4 * a * c

# If the discriminant is zero or something else, can't proceed.

if (disc > 0) {

out <- disc

} else if (disc == 0) {

# msg <- "There is only one real solution for the Johnson-Neyman interval.

# Values cannot be supplied."

# warning(msg)

return(NULL)

} else {

# msg <- "There are no real solutions for the Johnson-Neyman interval.

# Values cannot be supplied."

# warning(msg)

return(NULL)

}

return(out)

}

disc <- discriminant(a,b,c)

# Create value for attribute containing info on success/non-success of

# finding j-n interval analytically

if (is.null(disc)) {

failed <- TRUE

} else {

failed <- FALSE

}

# As long as the above didn't error, let's solve the quadratic with

# this function

quadsolve <- function(a,b,c, disc) {

# first return value

x1 <- (-b + sqrt(disc)) / (2 * a)

# second return value

x2 <- (-b - sqrt(disc)) / (2 * a)

# return a vector of both values

result <- c(x1, x2)

# make sure they are in increasing order

result <- sort(result, decreasing = FALSE)

return(result)

}

if (!is.null(disc)) {

bounds <- quadsolve(a,b,c, disc)

} else {

bounds <- c(-Inf, Inf)

}

names(bounds) <- c("Lower", "Higher")

# Need to calculate confidence bands

cbands <- function(x2, y1, y3, covy1, covy3, covy1y3, tcrit, predl, modx) {

upper <- c() # Upper values

slopes <- c() # predicted slope line

lower <- c() # Lower values

# Iterate through mod values given

slopesf <- function(i) {

# Slope

s <- y1 + y3*i

return(s)

}

upperf <- function(i, s) {

# Upper confidence band

u <- s + tcrit * sqrt((covy1 + 2*i*covy1y3 + i^2 * covy3))

return(u)

}

lowerf <- function(i, s) {

# Lower confidence band

l <- s - tcrit * sqrt((covy1 + 2*i*covy1y3 + i^2 * covy3))

return(l)

}

slopes <- sapply(x2, slopesf, simplify = "vector", USE.NAMES = FALSE)

upper <- mapply(upperf, x2, slopes)

lower <- mapply(lowerf, x2, slopes)

out <- matrix(c(x2, slopes, lower, upper), ncol = 4)

colnames(out) <- c(modx, predl, "Lower", "Upper")

out <- as.data.frame(out)

return(out)

}

# Generating values to feed to the CI function from the range

x2 <- seq(from = modrange[1], to = modrange[2], length.out = 1000)

# Make slopes colname

predl <- paste("Slope of", pred)

cbs <- cbands(x2, y1, y3, covy1, covy3, covy1y3, tcrit, predl, modx)

out$bounds <- bounds

out <- structure(out, modrange = modrangeo)

# Need to check whether sig vals are within or outside bounds

sigs <- which((cbs$Lower < 0 & cbs$Upper < 0) |

(cbs$Lower > 0 & cbs$Upper > 0))

# Going to split cbands values into significant and insignificant pieces

insigs <- setdiff(1:1000, sigs)

# Create grouping variable in cbs

cbs$Significance <- rep(NA, nrow(cbs))

cbs$Significance <- factor(cbs$Significance, levels = c("Insignificant",

"Significant"))

index <- 1:1000 %in% insigs

cbs$Significance[index] <- "Insignificant"

index <- 1:1000 %in% sigs

cbs$Significance[index] <- "Significant"

# Give user this little df

out$cbands <- cbs

# I'm looking for whether the significant vals are inside or outside

## Would like to find more elegant way to do it

index <- which(cbs$Significance == "Significant")[1]

if (!is.na(index) & index != 0) {

inside <- (cbs[index,modx] > bounds[1] && cbs[index,modx] < bounds[2])

all_sig <- NULL # Indicator for whether all values are either T or F

# We don't know from this first test, so we do another check here

if (is.na(which(cbs$Significance == "Insignificant")[1])) {

all_sig <- TRUE

} else {

all_sig <- FALSE

}

} else {

inside <- FALSE

all_sig <- TRUE

}

out <- structure(out, inside = inside, failed = failed, all_sig = all_sig)

# Splitting df into three pieces

cbso1 <- cbs[cbs[,modx] < bounds[1],]

cbso2 <- cbs[cbs[,modx] > bounds[2],]

cbsi <- cbs[(cbs[,modx] > bounds[1] & cbs[,modx] < bounds[2]),]

# Create label based on alpha level

alpha <- alpha * 2 # Undoing what I did earlier

alpha <- gsub("0\\.", "\\.", as.character(alpha))

pmsg <- paste("p <", alpha)

# Let's make a J-N plot

plot <- ggplot2::ggplot() +

ggplot2::geom_ribbon(data = cbso1,

ggplot2::aes(x = cbso1[,modx], ymin = cbso1[,"Lower"],

ymax = cbso1[,"Upper"],

fill = cbso1[,"Significance"]),

alpha = 1) +

ggplot2::geom_ribbon(data = cbsi,

ggplot2::aes(x = cbsi[,modx], ymin = cbsi[,"Lower"],

ymax = cbsi[,"Upper"],

fill = cbsi[,"Significance"]),

alpha = 1) +

ggplot2::geom_ribbon(data = cbso2,

ggplot2::aes(x = cbso2[,modx], ymin = cbso2[,"Lower"],

ymax = cbso2[,"Upper"],

fill = cbso2[,"Significance"]),

alpha = 1) +

ggplot2::geom_path(data = cbso1, ggplot2::aes(x = cbso1[,modx],

y = cbso1[,predl]),

size = line.thickness) +

ggplot2::geom_path(data = cbsi,

ggplot2::aes(x = cbsi[,modx], y = cbsi[,predl]),

size = line.thickness) +

ggplot2::geom_path(data = cbso2,

ggplot2::aes(x = cbso2[,modx], y = cbso2[,predl]),

size = line.thickness) +

ggplot2::scale_fill_manual(values = c("Significant" = sig.color,

"Insignificant" = insig.color),

labels = c("n.s.", pmsg),

breaks = c("Insignificant","Significant"),

drop = FALSE,

guide = ggplot2::guide_legend(order = 2)) +

ggplot2::geom_hline(ggplot2::aes(yintercept = 0))

if (is.null(mod.range)) {

plot <- plot +

ggplot2::geom_segment(ggplot2::aes(x = modrangeo[1], xend = modrangeo[2],

y = 0, yend = 0,

linetype = "Range of\nobserved\ndata"),

lineend = "square", size = 1.25)

}

# Adding this scale allows me to have consistent ordering

plot <-

plot +

ggplot2::scale_linetype_discrete(

name = " ", guide = ggplot2::guide_legend(order = 1)

)

if (out$bounds[1] < modrange[1]) {

# warning("The lower bound is outside the range of the plotted data")

} else if (all_sig == FALSE) {

plot <- plot +

ggplot2::geom_vline(ggplot2::aes(xintercept = out$bounds[1]),

linetype = 2, color = sig.color)

}

if (out$bounds[2] > modrange[2]) {

# warning("The upper bound is outside the range of the plotted data")

} else if (all_sig == FALSE) {

plot <- plot +

ggplot2::geom_vline(ggplot2::aes(xintercept = out$bounds[2]),

linetype = 2, color = sig.color)

}

plot <- plot + ggplot2::xlim(range(cbs[,modx])) +

ggplot2::labs(title = title, x = modx, y = predl) +

ggplot2::scale_color_manual(name = "",

values = c("Significant" = sig.color,

"Insignificant" = insig.color), guide = "none") +

jtools::theme_apa(legend.pos = "right", legend.font.size = 10) +

ggplot2::theme(legend.key.size = ggplot2::unit(1, "lines"))

out$plot <- plot

class(out) <- "johnson_neyman"

return(out)

}

J_N_IR <- johnson_neyman_blavaan(model=fit_model2_final, data = d_between, pred ="CA_Slope_c", pred2 = "b2",

modx = "BO_EE_Mean_c", modx2 = "d2", intterm = "BO_EE_Mean_c:CA_Slope_c",

intterm2 ="m2", outcome ="IR_Slope",

sig.color = "grey70",

insig.color = "grey90",

mod.range = c(-1.67, 3.33), title ="")

J_N_FH <- johnson_neyman_blavaan(model=fit_model2_final, data = d_between, pred ="CA_Slope_c", pred2 = "b1",

modx = "BO_EE_Mean_c", modx2 = "d1", intterm = "BO_EE_Mean_c:CA_Slope_c",

intterm2 ="m1", outcome ="FH_Slope",

sig.color = "grey70",

insig.color = "grey90",

mod.range = c(-1.67, 3.33), title ="")

J_N_IR$bounds

J_N_FH$bounds

# Modify and save graphs

png(filename = "C:/Users/Hohnemann/sciebo/04 Paper/04 MBSR/JNP_Stress.png", width = 500, height = 250)

J_N_IR$plot +

labs(title = "",

x = "Emotional Exhaustion (centered)",

y = "Moderated effect of ∆ Mindfulness\n on ∆ Stress") +

theme(text = element_text( family="Times New Roman"), axis.title= element_text(family="Times New Roman"))

graphics.off()

png(filename = "C:/Users/Hohnemann/sciebo/04 Paper/04 MBSR/JNP_Flow.png", width = 500, height = 250)

J_N_FH$plot +

labs(title = "",

x = "Emotional Exhaustion (centered)",

y = "Moderated effect of ∆ Mindfulness\n on ∆ Flow Experience") +

theme(text = element_text( family="Times New Roman"), axis.title= element_text(family="Times New Roman"))

graphics.off()

#### WAMBS Checklist for the mixed models obtaining change trajectories (random intercept, random slope) ######

library (ggplot2)

## 1.Do you understand the priors?

# non-informative default priors are used

## 2. Does the trace-plot exhibit convergence?

# Choose one outcome and run the commands below

model <- fm1_r # mindfulness

model <- fm2_r # flow

model <- fm3_r # stress

#run these to check for convergence

modeltranformed <- ggs(model)

ggplot(filter(modeltranformed, Parameter %in% c("b_Intercept", "b_Fragebogen", "sigma"),

Iteration > 1000),

aes(x = Iteration,

y = value,

col = as.factor(Chain)))+

geom_line()+

facet_grid(Parameter ~ .,

scale = 'free_y',

switch = 'y')+

labs(title = "Caterpillar Plots",

col = "Chains")+

theme_bw()

# Alternative: mcmc_plot(model, type = "trace")

# Gelman and Rubin diagnostic

modelposterior <- as.mcmc(model) # with the as.mcmc() command we can use all the CODA package convergence statistics and plotting options

gelman.diag(modelposterior[, 1:4])

gelman.plot(modelposterior[, 1:4])

geweke.diag(modelposterior[, 1:2])

geweke.plot(modelposterior[, 1:2])

## 3. Does convergence remain after doubling the number of iterations?

fm1_r_di <- brm(CA_Mean ~ Fragebogen + 1 + (Fragebogen|SERIAL), Dat2, warmup = 2000, iter = 20000, cores = 8)

fm2_r_di <- brm(FH_Mean ~ Fragebogen + 1 + (Fragebogen|SERIAL), Dat2, warmup = 2000, iter = 20000, cores = 8)

fm3_r_di <- brm(IR_Mean ~ Fragebogen + 1 + (Fragebogen|SERIAL), data = Dat2, control = list(adapt_delta = .99), warmup = 2000, iter = 20000, cores = 8) # increases adapt_delta beacuse of divergent interations

# Choose one outcome and check graphs and statistics above

model_di <- fm1_r_di # mindfulness

model <- fm1_r # mindfulness

model_di <- fm2_r_di # flow

model <- fm2_r # flow

model_di <- fm3_r_di # stress

model <- fm3_r # stress

modelposterior_di <- as.mcmc(model_di) # with the as.mcmc() command we can use all the CODA package convergence statistics and plotting options

gelman.diag(modelposterior_di[, 1:4])

geweke.diag(modelposterior_di[, 1:2])

# compute relative deviation

round(100*((summary(model_di)$fixed - summary(model)$fixed) / summary(model)$fixed), 3)[,"Estimate"]

## 4. Does the posterior distribution histogram have enough information?

mcmc_plot(fm1_r, type = "hist")

mcmc_plot(fm2_r, type = "hist")

mcmc_plot(fm3_r, type = "hist")

## 5. Do the chains exhibit a strong degree of autocorrelation?

modelposterior1 <- as.mcmc(fm1_r) # with the as.mcmc() command we can use all the CODA package convergence statistics and plotting options

modelposterior2 <- as.mcmc(fm2_r) # with the as.mcmc() command we can use all the CODA package convergence statistics and plotting options

modelposterior3 <- as.mcmc(fm3_r) # with the as.mcmc() command we can use all the CODA package convergence statistics and plotting options

autocorr.diag(modelposterior1[,1:5], lags = c(0, 1,2,3,4, 5, 10, 50))

autocorr.diag(modelposterior2[,1:5], lags = c(0, 1,2,3,4, 5, 10, 50))

autocorr.diag(modelposterior3[,1:5], lags = c(0, 1,2,3,4, 5, 10, 50))

## 6. Do the posterior distributions make substantive sense?

model <- fm1_r # mindfulness

model <- fm2_r # flow

model <- fm3_r # stress

modeltranformed <- ggs(model)

ggplot(filter(modeltranformed, Parameter %in% c("b_Intercept", "b_Fragebogen"),

Iteration > 1000),

aes(x = value,

fill = Parameter))+

geom_density(alpha = .5)+

geom_vline(xintercept = 0,

col = "grey",

size = 1)+

scale_x_continuous(name = "Value",

limits = c(-0.5, 4.5))+

geom_vline(xintercept = summary(model)$fixed[1,3], col = "blue", linetype = 2)+

geom_vline(xintercept = summary(model)$fixed[1,4], col = "blue", linetype = 2)+

geom_vline(xintercept = summary(model)$fixed[2,3], col = "red", linetype = 2)+

geom_vline(xintercept = summary(model)$fixed[2,4], col = "red", linetype = 2)+

theme_light()+

scale_fill_manual(name = 'Parameters',

values = c("red", "lightblue"),

labels = c(expression( " " ~ gamma[Time]),

expression( " " ~ gamma[Intercept])))+

labs(title = "Posterior Density of Parameters With 95% CCI lines")

## 7. Do different specification of the multivariate variance priors influence the results?

get_prior(CA_Mean ~ Fragebogen + 1 + (Fragebogen|SERIAL), Dat2)

PRIORS <- c(set_prior("normal(0,2)", class = "b", coef= "Fragebogen"), # coefficient Time

set_prior("cauchy(0,2)", class = "sd"), # a half cauchy distribution (truncuated at 0) for the sd

set_prior("lkj(2)", class = "cor"), # a Cholesky of 2 for the correlation

set_prior("inv_gamma(.5,.5)", class = "sigma")) # an uniformative inverse gamma for the sigma.

modeldifferentMVpriors_1 <- brm(CA_Mean ~ Fragebogen + 1 + (Fragebogen|SERIAL),

data = Dat2,

warmup = 1000,

iter = 10000,

chains = 4,

prior = PRIORS,

sample_prior = TRUE,

cores = 4) # the cores function tells STAN to make use of 4 CPU cores simultaneously instead of just 1.

modeldifferentMVpriors_2 <- brm(FH_Mean ~ Fragebogen + 1 + (Fragebogen|SERIAL),

data = Dat2,

warmup = 1000,

iter = 10000,

chains = 4,

prior = PRIORS,

sample_prior = TRUE,

cores = 4) # the cores function tells STAN to make use of 4 CPU cores simultaneously instead of just 1.

modeldifferentMVpriors_3 <- brm(IR_Mean ~ Fragebogen + 1 + (Fragebogen|SERIAL),

data = Dat2,

warmup = 1000,

iter = 10000,

chains = 4,

control = list(adapt_delta = 0.99),

prior = PRIORS,

sample_prior = TRUE,

cores = 4) # the cores function tells STAN to make use of 4 CPU cores simultaneously instead of just 1.

summary(fm1_r)

summary(modeldifferentMVpriors_1)

summary(fm2_r)

summary(modeldifferentMVpriors_2)

summary(fm3_r)

summary(modeldifferentMVpriors_3)

round(100*((summary(modeldifferentMVpriors_1)$fixed - summary(fm1_r)$fixed) / summary(fm1_r)$fixed), 3)[,"Estimate"]

round(100*((summary(modeldifferentMVpriors_2)$fixed - summary(fm2_r)$fixed) / summary(fm2_r)$fixed), 3)[,"Estimate"]

round(100*((summary(modeldifferentMVpriors_3)$fixed - summary(fm3_r)$fixed) / summary(fm3_r)$fixed), 3)[,"Estimate"]

## 8. Is there a notable effect of the prior when compared with non-informative priors?

## not applicable

## 9. Are the results stable from a sensitivity analysis?

## not applicable

## 10. Is the Bayesian way of interpreting and reporting model results used?

## no statistical calculation required

#### WAMBS Checklist for path models ######

## 1. Do you understand the priors?

# non-informative default priors are used

## 2. Does the trace-plot exhibit convergence?

plot(fit_model1_final, pars = 1:12, plot.type = "trace")

plot(fit_model2_final, pars = 1:14, plot.type = "trace")

mcmc.list <- blavInspect(fit_model1_final, what = "mcmc")

gelman.diag(mcmc.list)

gelman.plot(mcmc.list)

geweke.diag(mcmc.list)

geweke.plot(mcmc.list)

mcmc.list <- blavInspect(fit_model2_final, what = "mcmc")

gelman.diag(mcmc.list)

gelman.plot(mcmc.list)

geweke.diag(mcmc.list)

geweke.plot(mcmc.list)

## 3. Does convergence remain after doubling the number of iterations?

library(blavaan)

fit_model1_final_di <- bsem(model1_final, data = d_between, bcontrol=list(cores=8), burnin = 2000, sample =12000)

fit_model2_final_di <- bsem(model2_final, data = d_between, bcontrol=list(cores=8), burnin = 2000, sample =12000)

## Model 1

plot(fit_model1_final_di, pars = 1:12, plot.type = "trace")

mcmc.list <- blavInspect(fit_model1_final_di, what = "mcmc")

gelman.diag(mcmc.list)

gelman.plot(mcmc.list)

geweke.diag(mcmc.list)

geweke.plot(mcmc.list)

# compute relative deviation

estimates <- blavInspect(fit_model1_final, what = "postmean")[1:12]

estimates_di <- blavInspect(fit_model1_final_di, what = "postmean")[1:12]

round(100*(estimates_di - estimates) / estimates, 2)

## Model 2 di

plot(fit_model2_final_di, pars = 1:14, plot.type = "trace")

mcmc.list <- blavInspect(fit_model2_final_di, what = "mcmc")

gelman.diag(mcmc.list)

gelman.plot(mcmc.list)

geweke.diag(mcmc.list)

geweke.plot(mcmc.list)

# compute relative deviation

estimates <- blavInspect(fit_model2_final, what = "postmean")[1:14]

estimates_di <- blavInspect(fit_model2_final_di, what = "postmean")[1:14]

round(100*(estimates_di - estimates) / estimates, 2)

## 4. Does the posterior distribution histogram have enough information?

par(mfrow = c(4,4))

plot(fit_model2_final , pars = 1:14, plot.type = "hist")

par(mfrow = c(3,4))

plot(fit_model1_final , pars = 1:12, plot.type = "hist")

## 5. Do the chains exhibit a strong degree of autocorrelation?

par(mfrow = c(4,4))

plot(fit_model2_final, pars = 1:14, plot.type = "acf")

par(mfrow = c(3,4))

plot(fit_model1_final, pars = 1:12, plot.type = "acf")

## 6. Do the posterior distributions make substantive sense?

par(mfrow = c(4,4))

plot(fit_model2_final, pars = 1:14, plot.type = "dens")

par(mfrow = c(3,4))

plot(fit_model1_final, pars = 1:12, plot.type = "dens")

## 7. Do different specification of the multivariate variance priors influence the results?

dpriors(target = "stan")

mydp <- dpriors(alpha ="normal(0,5)", lambda="normal(0,5)", beta="normal(0,5)",theta ="gamma(1.5,.1)[sd]",psi ="gamma(1.5,.1)[sd]" )

fit_model1_final_DP <- bsem(model1_final, dp = mydp, data = d_between, bcontrol=list(cores=8), burnin = 2000, sample = 6000)

summary(fit_model1_final_DP, rsquare=T, nd =5)

# compute relative deviation

estimates_DP <- blavInspect(fit_model1_final_DP, what = "postmean")[1:14]

estimates <- blavInspect(fit_model1_final, what = "postmean")[1:14]

round(100*(estimates_DP - estimates) / estimates, 2)

fit_model2_final_DP <- bsem(model2_final, dp = mydp, data = d_between, bcontrol=list(cores=8), burnin = 2000, sample = 6000)

summary(fit_model2_final_DP, rsquare=T, nd =5)

# compute relative deviation

estimates_DP <- blavInspect(fit_model2_final_DP, what = "postmean")[1:14]

estimates <- blavInspect(fit_model2_final, what = "postmean")[1:14]

round(100*(estimates_DP - estimates) / estimates, 2)

## 8. Is there a notable effect of the prior when compared with non-informative priors?

## not applicable

## 9. Are the results stable from a sensitivity analysis?

## not applicable

## 10. Is the Bayesian way of interpreting and reporting model results used?

## no statistical calculation required
